# A software tool to automatically assure and report daily treatment deliveries by a cobalt‐60 radiation therapy device

**DOI:** 10.1120/jacmp.v17i3.6001

**Published:** 2016-05-08

**Authors:** Deshan Yang, H. Omar Wooten, Olga Green, Harold H. Li, Shi Liu, Xiaoling Li, Vivian Rodriguez, Sasa Mutic, Rojano Kashani

**Affiliations:** ^1^ Department of Radiation Oncology School of Medicine, Washington University in St. Louis St Louis MO USA

**Keywords:** Radiation therapy, quality assurance, image‐guidance

## Abstract

The aims of this study were to develop a method for automatic and immediate verification of treatment delivery after each treatment fraction in order to detect and correct errors, and to develop a comprehensive daily report which includes delivery verification results, daily image‐guided radiation therapy (IGRT) review, and information for weekly physics reviews. After systematically analyzing the requirements for treatment delivery verification and understanding the available information from a commercial MRI‐guided radiotherapy treatment machine, we designed a procedure to use 1) treatment plan files, 2) delivery log files, and 3) beam output information to verify the accuracy and completeness of each daily treatment delivery. The procedure verifies the correctness of delivered treatment plan parameters including beams, beam segments and, for each segment, the beam‐on time and MLC leaf positions. For each beam, composite primary fluence maps are calculated from the MLC leaf positions and segment beam‐on time. Error statistics are calculated on the fluence difference maps between the plan and the delivery. A daily treatment delivery report is designed to include all required information for IGRT and weekly physics reviews including the plan and treatment fraction information, daily beam output information, and the treatment delivery verification results. A computer program was developed to implement the proposed procedure of the automatic delivery verification and daily report generation for an MRI guided radiation therapy system. The program was clinically commissioned. Sensitivity was measured with simulated errors. The final version has been integrated into the commercial version of the treatment delivery system. The method automatically verifies the EBRT treatment deliveries and generates the daily treatment reports. Already in clinical use for over one year, it is useful to facilitate delivery error detection, and to expedite physician daily IGRT review and physicist weekly chart review.

PACS number(s): 87.55.km

## I. INTRODUCTION

The MRIdian magnetic resonance image‐guided radiation therapy (MR‐IGRT) system by ViewRay (ViewRay Inc., Cleveland, OH) is one of the newest significant developments for radiation cancer treatment.^1^, ^2^ It combines real‐time magnetic resonance (MR) image guidance^3^, ^4^ and intensity‐modulated radiation therapy (IMRT^(5)^) technologies to allow soft tissue visualization, accurate tumor targeting, and simultaneous radiation delivery. In addition, the MRIdian has the capability of online treatment plan adaptation^6^, ^7^ based on daily volumetric MR imaging; therefore, it could optimize the patient radiation treatment plan by adapting to the patient anatomy status of the day. Such important treatment plan adaptation function could potentially maximize the treatment accuracy while minimizing the toxicities to organs‐at‐risk surrounding the treatment target.

MRIdian was at its very early stage of clinical implementation at the beginning of this study, in which we aimed to address two important tasks — automatic daily treatment delivery verification and daily treatment reporting. These tasks are important because they are directly related to patient safety, quality assurance, and workflow efficiency.

A treatment delivery report serves multiple purposes. It allows the radiation therapists to quickly check the accuracy and completeness of the treatment deliveries. In addition, it allows radiation oncologists to quickly verify the image‐guided patient setups and the overall course of patient treatments. It also allows medical physicists to perform patient chart checks quickly to ensure the accuracies of the patient treatment delivery.^(8)^ To maximize efficiency and the responsiveness of error detection, the treatment delivery reports should be generated quickly and automatically immediately after each treatment delivery without requiring manual work by therapists. The report should include all the information that is required for therapists', physicians', and physicists' reviews, and should be concise and comprehensive so that the report can be checked not only quickly, but also more effectively.

The previous MRIdian reports were insufficient to support all these purposes. We were therefore motivated to redesign the report and to include the machine log‐based delivery checks. Our overall idea was 1) to obtain the treatment machine log files, which are automatically generated during each treatment delivery; 2) to analyze and compare the log files against the treatment plans in order to check the completeness and accuracy of the treatment deliveries; and 3) to generate concise and comprehensive reports.

Methods for analysis and presentation of machine log files have been previously reported for linear accelerator‐(linac‐) based external beam radiation therapy (EBRT) treatments.^9^, ^10^, ^11^, ^12^, ^13^ The MLC dynamic log files are available on Varian linac treatment machines^(14)^ and have been utilized at the authors' institution and by other groups to validate the accuracy and completeness of the treatment deliveries.^15^, ^16^, ^17^, ^18^, ^19^, ^20^, ^21^ Based on our previous experience, we developed a new treatment delivery verification procedure for ViewRay in this study. The main features included: 1) verification of key treatment delivery parameters (obtained from the treatment delivery log files) against the approved treatment plan, and 2) comprehensive comparison of beam delivery parameters using 2D fluence maps. A computer program, named VRDCR (ViewRay Delivery Check and Report), was developed to implement the procedure to automatically perform the delivery verification and generate the reports. VRDCR was fully tested and integrated into the ViewRay treatment delivery system. The new treatment delivery report allows therapists, physicians, and medical physicists to quickly and efficiently verify the accuracy of the image guidance and treatment deliveries.

## II. MATERIALS AND METHODS

### A. Workflow

**Figure 1:**
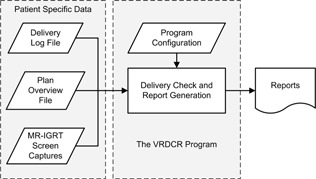
The system workflow.

The simplified system workflow is shown in Fig. 1. The VRDCR program is designed to be simple and requires no manual interventions so that it can be quickly invoked immediately following completion of each fraction. It will automatically import and process the patient‐specific data provided to it, perform the checks, and generate the report.

### B. Materials

The MRIdian system has three cobalt‐60 treatment heads, 120° apart, with each providing a nominal dose rate, 1.85 Gy/min at the new source installation. The three heads together provide a total dose rate comparable to that of a conventional linear accelerator (linac) using simultaneous delivery. Treatment plans are created in the ViewRay TPS. Each plan contains multiple treatment beam groups (i.e., gantry positions) each of which contains one to three beams. Beams belonging to the same beam groups have gantry angles 120° apart and therefore could be delivered simultaneously by three treatment heads. Each beam contains one or multiple segments. Each segment is defined by a MLC formed beam aperture and a beam‐on time. There are total of 60 MLC leaves in 30 pairs. Each individual beam is delivered in the step‐and‐shoot method. Radiation will be turned on for delivering one beam segment at a time. Between segments, the radiation will be turned off (the cobalt source will be moved to the off position) and the MLC leaves will move to the next position.

The patient‐specific data used by VRDCR are the treatment delivery log file, the plan overview file, and image‐guided patient setup screen capture files. The plan data provided by the ViewRay system is the plan overview file, which contains all the basic treatment plan information — patient name, ID, plan name, date, prescription name, prescription dose, planning target volume (PTV) name, and the treatment fraction configuration. The plan overview file also contains information on the entire treatment plan, including the beam parameters (gantry angle, number of segments) and segment parameters (MLC positions, beam‐on time). ViewRay uses the step‐and‐shoot method for IMRT delivery. Each IMRT plan contains multiple treatment beams at different gantry angle positions, and each beam contains multiple segments. The plan overview file is a text format file which can be manually exported from ViewRay TPS, or provided to VRDCR automatically by the ViewRay treatment machine at the end of a treatment delivery.

A single treatment delivery log file is generated automatically by the ViewRay treatment machine at the end of every treatment delivery. In addition to the basic plan information (e.g., patient name, ID, plan name, and fraction number), the log file also contains the actual treatment beam parameters that are recorded during the treatment delivery, including gantry angle, MLC leaf positions of each segment, beam‐on and beam‐off time of each beam segment, and the cobalt‐60 source strength and dose rate information of the treatment day. It is important to note that the per‐segment beam‐on times provided in the plan overview file are defined at the treatment planning system's nominal planning dose rate of 1.85 Gy/min, and the beam‐on times recorded in the log file are the as‐delivered beam‐on times based on the decayed dose rate for each of the three cobalt‐60 sources on the treatment day.

The most significant feature of MRIdian over linac‐based EBRT is MR image guidance, which allows soft tissue visualization for daily localization and real‐time motion management.

In our institution, physicians do not need to be present at the treatment machine for every treatment fraction. They are, however, required to check the daily MR‐IGRT patient setup by reviewing the patient daily setup images, or in the case of ViewRay, the patient setup screen capture image. Due to the limited implementation of DICOM protocols, ViewRay system is not integrated with MOSAIQ (Elekta, Stockholm, Sweden), the treatment management system (TMS) used at the authors' institution. The 3D daily MR images and the corresponding image registration parameters could be reviewed in the MRIdian TPS but not in MOSAIQ as most linac machines do. To allow the patient setup image review in MOSAIQ by the physicians (a routine step in the physicians' clinical workflow at the authors' institution), the patient setup screen capture images are acquired by MRIdian automatically at the time when the therapists confirm the patient alignment after the daily MRI image and the treatment planning images are manually registered. These screen capture images are provided to the VRDCR program, which does not check these images, but simply inserts them into the treatment delivery report. The final report in PDF format is manually loaded into MOSAIQ by the therapist as a patient document.

### C. Implementation

**Figure 2:**
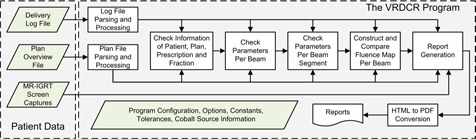
VRDCR program flow chart.

A more detailed workflow of the VRDCR program is shown in Fig. 2. Specifically, VRDCR performs the delivery verification and report generation in the following steps:

1. Parse and convert the plan overview file and the log file into composite data structures so that the data elements can be utilized by the program code.
2. Check the patient, plan, and prescription information listed in Table 1.3.
3. Check each beam for the parameters listed in Table 1.4.
4. Check each beam segment for the parameters listed in Table 1.5.
5. For each beam, construct and check the integrated primary fluence map.
6. Check the cobalt‐60 source information in the log file against the respective data in the VRDCR configuration XML file, which also contains program configuration options, the tolerance values, and other global constants (e.g., the nominal source strength used in the treatment planning).

**Table 1:** List of items in categories checked by VRDCR program.

*Categories*	*Items*
Patient information	Patient name and ID
Prescription information	Prescription dose, PTV target, number of treatment fractions
Cobalt‐60 source information	Source serial #, calibration date, calibration source strength and dose rate, the decayed source strength and dose rate
Treatment plan	Plan name, total number of treatment beams
Per beam	Gantry angle, number of segments, total beam‐on time, beam fluence intensity map
Per segment	MLC leaf positions, beam‐on time

Most items in the treatment delivery log files are checked against the respective items in the plan overview file. The cobalt‐60 source information is checked against the data entered in the VRDCR configuration XML file because the source information is not available in the plan overview file. The beam primary fluence intensity maps are computed by integrating the beam aperture multiplied by beam‐on time, per beam segment, as:

(1) $$F(x,y)=\sum_{k=1}^{N}t_{k}A_{k}(x,y)$$

where F(x,y) is the 2D beam fluence map at the machine isocenter on the 2D plane perpendicular to the beam direction, *x* and *y* are the 2D coordinates on the isocenter plane, *k* is the beam segment number, *N* is the total number of beam segments, $t_{k}$ is the beam‐on time (in seconds) for the beam segment k, and $A_{k}(x,y)$ is the 2D beam aperture map at the isocenter plan. $A_{k}(x,y)=0$ if the point (x,y) is outside the beam aperture. For a point at (x,y), $A_{k}(x,y)$ can be determined directly from the MLC leaf position values for the beam segment k^15^. F(x,y) is in seconds.

Fluence maps are calculated separately with beam parameters in the treatment plan and in the delivery log. The mean, maximum, and standard deviation (SD) values of the fluence map difference are computed. Each pixel of the fluence map with an intensity value greater than 10% of the maximal intensity of the planned fluence map is considered as pass if the fluence difference on the pixel is less than 2% of the maximal value. Pixels with intensity values less than 10% of the maximal value are ignored. The parameter 2% is chosen empirically and a pass rate of 90% or higher is designated as acceptable.

Our current VRDCR program was programmed in MATLAB (MathWorks Inc., Natick, MA). There are two versions. The command line version is integrated into the ViewRay system at the treatment delivery console computer, which calls the VRDCR program when a delivery is finished and provides the plan overview file, the delivery log file, and the screen capture image files. The report is generated in HTML format and then automatically converted to the final PDF file. The font and style are controlled using a separated CSS file. A second version is a stand‐alone application with a simple graphic user interface. It is designed for QA staffs to create reports for the patient QA deliveries.

### D. Testing and clinical commissioning

We tested the VRDCR program and its functions extensively with the plan overview files and the delivery log files obtained from physics‐testing deliveries and real‐patient treatment deliveries, covering all combinations of treatment sites and treatment modalities. The error detection capabilities of VRDCR were verified with manually introduced delivery errors (e.g., treatment interruption, beam skipping, incorrect treatment plan version, delivery of treatment plan of a different patient), as well as data files with manually entered artificial errors (e.g., incorrect cobalt source decay information in the log file, MLC leaf position errors, wrong beam‐on and beam‐off time).

VRDCR was clinically commissioned after it was integrated in the ViewRay treatment delivery system. The system integration, the automatic generation of treatment delivery report, and the accuracy of the reports were confirmed and evaluated during the clinical commissioning process. In clinical commissioning, comprehensive tests were performed to investigate the VRDCR's sensitivity to delivery errors. A standard baseline, containing a single static field and three fields in a 3D conformal plan, was established. Variations of the baseline, which included rotated gantry, changed field size, shifted directions, and changed delivery time, were delivered and verified with VRDCR against the baseline.

## III. RESULTS

**Figure 3:**
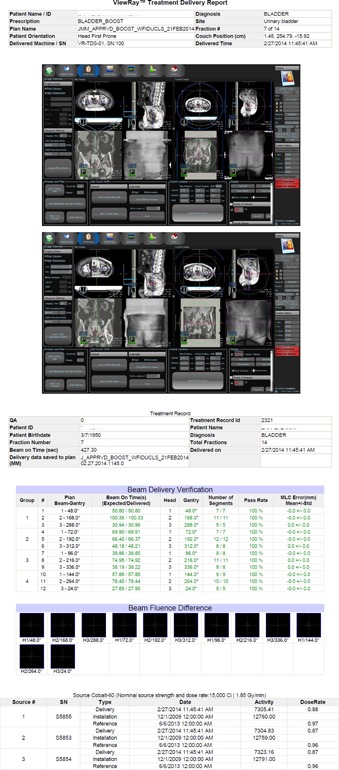
A patient treatment delivery report: (left) plan information and image guidance screen captures; (right) beam delivery verification results and the cobalt source information. The fluence difference maps are all black because there was no significant difference between the beam fluence computed from the planned beam parameters and the delivered beam parameters.

The VRDCR program was successfully implemented in MATLAB. It takes between 5 and 15 s, depended on the number of beams and beam segments in the plan, to perform delivery verification and report generation for each patient treatment delivery. Figure 3 shows an example of the generated report for a patient treatment delivery. The tolerance was set to 0.5° for gantry, 2 mm for MLC leaf positioning, and 0.2 s for beam‐on time, per considerations between the vendor specifications and AAPM TG‐142 recommendations. The fluence passing rate was defined as the percentage of the pixels with delivery errors less than 2% of the maximal fluence in the field. These tolerance values are user‐configurable in the VRDCR program configuration XML file.

VRDCR was developed prior to the clinical commissioning of the MRIdian system and was proven a useful physics tool before and during the clinical commissioning process. It was used to verify the treatment deliveries and to assess the system performance including the MLC leaf positional accuracy, daily dose rate computation accuracy, treatment delivery repeatability, and the correctness of interrupted and continued treatment deliveries. It was instrumental in identification of multiple minor system issues during earlier‐stage MRIdian system software updates and had allowed the issues to be fixed promptly by the vendor engineers before the new software releases were approved for clinical use.

**Figure 4:**
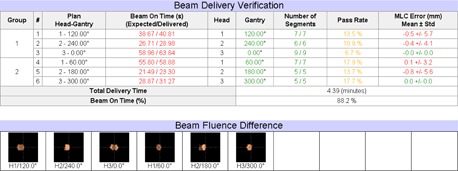
Demonstration of detected treatment delivery errors in an earlier software version test.

Figure 4 shows an example of detected errors in an earlier software version test in the year 2013. The errors were caused by incorrectly reported delivery beam‐on time in the log file, for which the beam‐on time after considering source‐strength decay should have been reported instead of the value before the source strength decay calculation. This error was confirmed by ViewRay and was fixed in the sequential software update. Other errors that were detected by VRDCR during system testing and commissioning included the incorrect positions of single closed MLC leaf pair (incorrect MLC leaf pair junction walk), beam‐on time difference over tolerance (the accumulated beam‐on time of the multiple beam segmentations of a single IMRT SMLC beam) due to numerical rounding applied in beam‐on time calculation, and inconsistencies in delivery log files (e.g., missing of beam segment identifiers).

**Figure 5:**
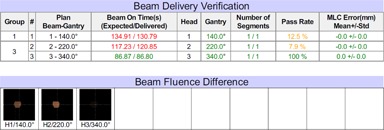
Example of the simulated errors detected by VRDCR in the clinical commissioning tests. In this example, the beam weighting was adjusted by 1% for beams 1 and 2. The errors were reflected as a change in the delivery beam‐on time, which also resulted in a difference in the beam fluence.

The clinical commissioning tests demonstrated that VRDCR was able to detect and report the simulated errors such as the gantry error greater than 0.5°, the beam weighting changed by greater than a percent (Fig. 5), a single MLC leaf error greater than 1 cm, and the daily prescription dose changed from 2 Gy to 1.8 Gy. VRDCR highlighted these differences that are greater than the institution‐defined tolerance in the report.

Because the ViewRay system is not integrated with MOSAIQ via DICOM protocols, treatment deliveries on ViewRay machine cannot be automatically recorded in MOSAIQ and verified in MOSAIQ afterward. Prior to VRDCR, there was no simple way for a physicist to verify the treatment deliveries, because the only treatment delivery records from ViewRay machine were the delivery log files. A physicist had to manually select and open each log file in a texteditor program, and visually compare the extensive content of the log files to the corresponding content in the treatment plan documentation. Such a manual process was very inefficient and not reliable. The treatment delivery verification reports generated by VRDCR have provided a means for the treatment deliveries to be recorded in MOSAIQ as PDF documents, and to allow the treatment deliveries to be quickly checked by medical physicists. It was roughly estimated that 10 min could have been saved for a physicist to verify five treatment deliveries (in one week) of a patient with the VRDCR reports versus with the raw treatment delivery log files.

## IV. DISCUSSION

The system developed herein enables the ViewRay system to verify the treatment delivery immediately after the completion of the treatment delivery, and allows the treatments to be reviewed by physicians and physicists faster and easier. The procedure and the computer program developed in this study allow a small workflow efficiency improvement. The delivery report allows a single stop for a physician to quickly check the daily IGRT patient setup, and for medical physicists to quickly check the treatment deliveries. It may be interesting to quantify such an efficiency improvement by comparing to the current treatment management system‐based clinical workflow.

We have also built a stand‐alone VRDCR program with a simple user interface for use at the time of patient‐specific pretreatment IMRT QA.^(22)^ The ViewRay treatment delivery log files are obtained from the treatment control computer immediately following the ArcCHECK (Sun Nuclear, Melbourne, FL) QA deliveries. The collected log files, and the treatment plan overview files that are exported by ViewRay TPS are imported into the stand‐alone ViewRay DQA program, in which the delivery logs are checked against the plans and the delivery verification reports are generated. The report PDF files are then imported into our clinical record and verify system, and are checked and approved by physicists before the treatment fractions are approved.

The procedure developed in this study can be adapted to linac‐based EBRT treatment. Linac machines by Varian generate treatment delivery log files (i.e., DynaLog files), that contain even more detailed information than the ViewRay machine. The daily CBCT images, 2D kV, and MV portal images, can be obtained in either the OBI (On‐Board Imager) computer of the treatment machine or the MOSAIQ computer next to the treatment machine. It is straightforward to improve our current automatic DynaLog QA programs 1) to check the DynaLog file (against the corresponding treatment plan in DICOM format) right after the treatment deliveries, and 2) to include the daily IGRT images, into a single delivery check report, similar to the ViewRay delivery verification report.

## V. CONCLUSIONS

A procedure was developed in this study and implemented in a computer program to automatically verify the ViewRay treatment deliveries and to generate concise daily treatment reports. The method is useful to facilitate delivery error detection, and to expedite physicians' daily IGRT review and physicists' weekly chart review.

## ACKNOWLEDGMENT

The project described was partially supported by the AHRQ (Agency for Healthcare Research and Quality) grant number 1 R01 HS022888‐01 and its contents are solely the responsibility of the authors and do not necessarily represent the official views of the Agency for Healthcare Research and Quality. This study is partially supported by a research grant from ViewRay Incorporated.

## COPYRIGHT

This work is licensed under a Creative Commons Attribution 4.0 International License.

## Supporting information

Supplementary Material
