# Supplementary material for: A software tool to automatically assure and report daily treatment deliveries by a cobalt‐60 radiation therapy device
Source: J Appl Clin Med Phys. 2016 May 8;17(3):492–501. doi: 10.1120/jacmp.v17i3.6001 (PMC5690925; doi:10.1120/jacmp.v17i3.6001)
Supplement: Supplementary file 1 — Supplementary Material [file ACM2-17-492-s001.docx]

A software tool to automatically assure and report external beam daily treatment deliveries for a MR image-guided radiation therapy device

# Abstract

**Purpose**: The aims of this study were to develop a method for automatic and immediate verification of treatment delivery after each treatment fraction in order to detect and correct errors, and to develop a comprehensive daily report which includes delivery verification results, daily image-guided radiation therapy (IGRT) review, and information for weekly physics reviews.

**Methods**: After systematically analyzing the requirements for treatment delivery verification and understanding the available information from a commercial MRI guided radiotherapy treatment machine, we designed a procedure to use 1) treatment plan files, 2) delivery log files, and 3) beam output information to verify the accuracy and completeness of each daily treatment delivery. The procedure verifies the correctness of delivered treatment plan parameters including beams, beam segments, and for each segment, the beam-on time and MLC leaf positions. For each beam, composite primary fluence maps are calculated from the MLC leaf positions and segment beam-on time. Error statistics are calculated on the fluence difference maps between the plan and the delivery. A daily treatment delivery report is designed to include all required information for IGRT and weekly physics reviews including the plan and treatment fraction information, daily beam output information, daily patient setup screen captures, and the treatment delivery verification results.

**Results**: A computer program was developed to implement the proposed procedure of the automatic delivery verification and daily report generation for a MRI guided radiation therapy system. The program was clinically commissioned. Sensitivity was measured with simulated errors. The final version has been integrated into the commercial version of the treatment delivery system.

**Conclusion**: A method was developed to automatically verify the EBRT treatment deliveries and generate the daily treatment reports. Already in clinical use for over one year, it is useful to facilitate the delivery error detection, and expedite physician daily IGRT review and physicist weekly chart review.

# Introduction

The MRIdian magnetic resonance image-guided radiation therapy (MR-IGRT) system by ViewRay (ViewRay Inc. Cleveland, OH) is one of the newest significant developments for radiation cancer treatment^1, 2^. It combines real-time magnetic resonance (MR) image guidance^3, 4^ and intensity modulated radiation therapy (IMRT^5^) technologies to allow soft tissue visualization, accurate tumor targeting, and simultaneous radiation delivery. In addition, the MRIdian has the capability of online treatment plan adaptation^6, 7^ based on daily volumetric MR imaging, and therefore could optimize the patient radiation treatment plan by adapting to the patient anatomy status of the day. Such important treatment plan adaption function could potentially maximize the treatment accuracy while minimizing the toxicities to organs-at-risks surrounding the treatment target.

MRIdian was at its very early stage of clinical implementation at the beginning of this study, in which we aimed to address two important tasks – automatic daily treatment delivery verification and daily treatment reporting. These tasks are important because they are directly related to patient safety, quality assurance, and workflow efficiency.

A treatment delivery report serves multiple purposes. It allows the radiation therapists to quickly check the accuracy and completeness of the treatment deliveries. It allows radiation oncologists to quickly verify the image guided patient setups and the overall course of patient treatments. It also allows medical physicists to perform patient chart checks quickly to ensure the accuracies of the patient treatment delivery^8^. To maximize efficiency and the responsiveness of error detection, the treatment delivery reports should be generated quickly and automatically immediately after each treatment delivery without requiring manual work by therapists. The report should include all the information that is required for therapists, physicians, and physicists reviews, and should be concise and comprehensive so that the reports can be checked not only quickly, but also more effectively.

The previous MRIdian reports were insufficient to support all these purposes. We were therefore motivated to redesign the report and to include the machine log-based delivery checks. Our overall idea was 1) to obtain the treatment machine log files, which are automatically generated during each treatment delivery, 2) to analyze and compare the log files against the treatment plans in order to check the completeness and accuracy of the treatment deliveries, and 3) to generate concise and comprehensive reports.

Methods for analysis and presentation of machine log files have been previously reported for linear accelerator (LINAC) based external beam radiation therapy (EBRT) treatments^9-13^. The MLC dynamic log files are available on Varian LINAC treatment machines^14^. At authors’ institution, we previously developed a computer program “DynaQA”, which stands for multi-leaf collimator (MLC) dynamic log file quality assurance (QA), or dynalog QA. To check the accuracy and completeness of the treatment deliveries, our DynaQA tool analyzes these log files and compares them to the treatment plan DICOM files in order to check the plan data transfer from the treatment planning system (TPS) to the record and verify system (R&V) and from there to the treatment machines. The DynaQA tool has been used in all pre-treatment patient specific QA at our institution since 2009. This tool was updated to support the TrueBeam log files in 2011^15^ and VMAT plans in 2012^16-19^. In 2011, we further developed the DynaQA tool into ADQ – automatic delivery QA^20, 21^. The ADQ programs runs automatically to perform delivery log file QA for all patient treatment deliveries at the end of a treatment day, not just the pre-treatment phantom QA deliveries. ADQ allows patient deliveries to be verified automatically without any additional costs. Similar efforts were also reported by other research groups^21^. ADQ was eventually licensed and is now a commercial product.

Based on our experience with these previous applications, we developed a new treatment delivery verification procedure for ViewRay in this study. The main features included: 1) verification of key treatment delivery parameters (obtained from the treatment delivery log files) against the approved treatment plan, 2) comprehensive comparison of beam delivery parameters using 2D fluence maps and 3) inclusion of the daily image guidance information in the delivery report. More importantly, this is, to our knowledge, the first delivery report in radiotherapy that includes daily analysis of planned and delivered treatment parameters, as well as the daily image guidance information. A computer program, named VRDCR (ViewRay Delivery Check and Report), was developed to implement the procedure to automatically perform the delivery verification and generate the reports. VRDCR was fully tested and integrated into the ViewRay treatment delivery system. The new treatment delivery report allows therapists, physicians and medical physicists to quickly and efficiently verify the accuracy of the image guidance and treatment deliveries.

# Method and Materials

## Workflow

The simplified system workflow is shown in Figure 1. The VRDCR program is designed to be simple and requires no manual interventions so that it can be quickly invoked immediately following completion of each fraction. It will automatically import and process the patient specific data provided to it, perform the checks, and generate the report.

## Materials

The MRIdian system has three Cobalt-60 treatment heads, 120º apart with each providing a nominal dose rate, 1.85 Gy/min at the new source installation. The three heads together provide a total dose rate comparable to that of conventional linear accelerator (LINAC) using simultaneous delivery. Treatment plans are created in the ViewRay TPS. Each plan contains multiple treatment beam groups, i.e. gantry positions, with each containing 1 to 3 beams. Beams belonging to the same beam groups have gantry angles 120º apart and therefore could be delivered simultaneously by three treatment heads. Each beam contains 1 or multiple segments. Each segment is defined by a MLC formed beam aperture and a beam-on time. There are totally 60 MLC leaves in 30 pairs. Each individual beam is delivered in the step-and-shot way. Radiation will be turned on for delivering one beam segment at a time. Between segments, the radiation will be turned off (the Cobalt source will be moved to the off position) and the MLC leaves will move to the next position.

The patient specific data used by VRDCR are the treatment delivery log file, the plan overview file and image-guided patient setup screen capture files. The plan data provided by the ViewRay system is the plan overview file, which contains all the basic treatment plan information, e.g. patient name, ID, plan name, date, prescription name, prescription dose, planning target volume (PTV) name, and the treatment fraction configuration. The plan overview file also contains information of the entire treatment plan, including the beam parameters (gantry angle, number of segments) and segment parameters (MLC positions, beam-on time). ViewRay uses step-and-shoot method for IMRT delivery. Each IMRT plan contains multiple treatment beams at different gantry angle position, and each beam contains multiple segments. The plan overview file is a text format file which can be manually exported from ViewRay TPS, or provided to VRDCR automatically by the ViewRay treatment machine at the end of a treatment delivery.

A single treatment delivery log file is generated automatically by the ViewRay treatment machine at the end of every treatment delivery. In addition to the basic plan information, e.g. patient name, ID, plan name, and fraction number, the log file also contains the actual treatment beam parameters that are recorded during the treatment delivery, including gantry angle, MLC leaf positions of each segment, beam-on and beam-off time of each beam segments, and the cobalt-60 source strength and dose rate information of the treatment day. It is important to note that the per-segment beam-on times provided in the plan overview file are defined at the treatment planning system’s nominal planning dose rate of 1.85 Gy/min, and the beam-on times recorded in the log file are the as-delivered beam-on times based on the decayed dose rate for each of the three cobalt-60 sources on the treatment day.

The most significant feature of MRIdian over LINAC-based EBRT is MR image guidance, which allows soft tissue visualization for daily localization and real-time motion management. In our institution, physicians do not need to be present at the treatment machine for every treatment fraction. They are, however, required to check the daily MR-IGRT patient setup by reviewing the patient daily setup images, or in the case of ViewRay, the patient setup screen capture image. The 3D daily MR images could be reviewed in the MRIdian TPS but MRIdian still cannot export the 3D daily MR images to the clinical R&V system as most LINAC machines do. To allow the patient setup image review in R&V by the physicians (a routine step in the physicians’ clinical workflow at authors’ institution), the patient setup screen capture images are acquired by MRIdian automatically at the time when the therapists confirm the patient alignment after the daily MRI image and the treatment planning images are manually registered. These screen capture images are provided to the VRDCR programs, which does not check these images, but simply inserts them into the treatment delivery report.

## Implementation

A more detailed workflow of the VRDCR program is shown in Figure 2. Specifically, VRDCR performs the delivery verification and report generation in the following steps:

1. Parse and convert the plan overview file and the log file into composite data structures so that the data elements can be utilized by the program code.
2. Check the patient, plan and prescription information listed in Table 1.
3. Check each beam for the parameters listed in Table 1.
4. Check each beam segment for the parameters listed in Table 1.
5. For each beam, construct and check the integrated primary fluence map.
6. Check the cobalt-60 source information in the log file against the respective data in the VRDCR configuration XML file, which also contains program configuration options, the tolerance values and other global constants, e.g. the nominal source strength used in the treatment planning.

Most items in the treatment delivery log files are checked against the respective items in the plan overview file. The cobalt-60 source information is checked against the data entered in the VRDCR configuration XML file because the source information is not available in the plan overview file. The beam primary fluence intensity maps are computed by integrating the beam aperture multiplied by beam-on time. Fluence maps are calculated independently with beam parameters in the treatment plan and in the delivery log. The mean, maximum, and standard deviation values of the fluence map difference are computed. A 2D gamma analysis is then applied to compute the gamma pass rate based on 2% dose-difference and 2 mm distance-to-agreement tolerance^22^.

Our current VRDCR program was programmed in MATLAB (Mathworks, Natick, MA). There are two versions. The command line version is integrated into the ViewRay system at the treatment delivery console computer, which calls the VRDCR program when a delivery is finished and provides the plan overview file, the delivery log file and the screen capture image files. The report is generated in HTML format and then automatically converted to the final PDF file. The font and style are controlled using a separated CSS file. A second version is a stand-alone application with a simple graphic user interface. It is designed for QA staffs to create reports for the patient QA deliveries.

## Testing and clinical commissioning

We tested the VRDCR program and its functions extensively with the plan overview files and the delivery log files obtained from physics testing deliveries and real patient treatment deliveries covering all combinations of treatment sites and treatment modalities. The error detection capabilities of VRDCR were verified with manually introduced delivery errors (e.g. treatment interruption, beam skipping, incorrect treatment plan version, delivery of treatment plan of a different patient), as well as data files with manually entered artificial errors (e.g. incorrect cobalt source decay information in the log file, MLC leaf position errors, wrong beam-on and beam-off time).

VRDCR was clinically commissioned after it was integrated in the ViewRay treatment delivery system. The system integration, the automatic generation of treatment delivery report, and the accuracy of the reports were confirmed and evaluated during the clinical commissioning process. In clinical commissioning, comprehensive tests were performed to investigate the VRDCR’s sensitivity to delivery errors. A standard baseline, containing a single static field and 3 fields in a 3D conformal plan, was established. Variations of the baseline, which included rotated gantry, changed field size, directional shifts, and changed delivery time, were delivered and verified with VRDCR against the baseline.

# Results

The VRDCR program was successfully implemented in MATLAB. It takes between 5 and 15 seconds, depended the number of beams and beam segments in the plan, to perform delivery verification and report generation for each patient treatment delivery. The command line version of VRDCR has been integrated into the official ViewRay treatment deliver system and clinically commissioned. A second version with a simple user interface, is also developed and tested for use by physics staff to verify the patient specific IMRT QA deliveries. Figure 3 shows an example of the generated report for a patient treatment delivery. The tolerance was set to 0.5 degree for gantry, 2 mm for MLC leaf positioning, and 0.2 sec for beam-on time. The fluence passing rate was defined as the percentage of the pixels with delivery errors less than 2% of the maximal fluence in the field. These tolerance values are user configurable in the VRDCR program configuration XML file.

VRDCR was developed prior to the clinical commissioning of the MRIdian system and was proven a useful physics tool before and during the clinical commissioning process. It was used to verify the treatment deliveries, to assess the system performance including the MLC leaf positional accuracy, daily dose rate computation accuracy, treatment delivery repeatability, the correctness of interrupted and continued treatment deliveries. It was instrumental in identification of multiple minor system issues during earlier stage MRIdian system software updates and had allowed the issues to be fixed promptly by the vendor engineers before the new software releases are approved for clinical use. For the example shown in Figure 4, VRDCR helped to detect the delivery errors in an earlier software version test in the year 2013. For this physical phantom beam delivery, VRDCR detected the beam-on time calculation errors and MLC position errors that were lately confirmed and fixed in the next official software release.

The clinical commissioning tests demonstrated that VRDCR was able to detect and report the simulated errors such as the gantry error greater than 0.5 degrees, the beam weighting changed by greater than a percent (Figure 5), a single MLC leaf error greater than 1cm, and the daily prescription dose changed from 2 Gy to 1.8 Gy. VRDCR highlighted these differences, that are greater than the institution-defined tolerance, in the report.

# Discussion

To our knowledge, the system developed herein is the first of its kind to enable an EBRT machine to verify the treatment delivery immediately after the completion of the treatment delivery, or to generate a comprehensive daily treatment report which contains all information required to serve both physician IGRT review and physics weekly chart review. This work allows the ViewRay treatment to be reviewed by physicians and physicists much faster and easier, compared to conventional LINAC treatment machines. This advantage could become more important in the next step ViewRay MRI guided online treatment adaptation.

The procedure and the computer program developed in this study allow a small workflow efficiency improvement. The delivery report allows a single stop for physician to quickly check the daily IGRT patient setup, and for medical physicists to quickly check the treatment deliveries. It may be interesting to quantify such an efficiency improvement by comparing to the current treatment management system based clinical workflow.

We have also built a stand-alone VRDCR program with simple user interface for use at the time of patient-specific pre-treatment IMRT QA^23^. The ViewRay treatment delivery log files are obtained from the ViewRay treatment control computer immediately following the treatment deliveries. The collected log files, and the treatment plan overview files that are exported by ViewRay TPS, are imported into the stand-alone ViewRay DQA program in which the delivery logs are checked against the plans and the delivery verification reports are generated. The report PDF files are then be imported into our clinical record and verify system, and are checked and approved by physicists before the treatment fractions are approved.

The procedure developed in this study can be adapted to LINAC-based EBRT treatment. LINAC machines by Varian generate treatment delivery log files (i.e. dynalog files), that contain even more detailed information than the ViewRay machine. The daily CBCT images, 2D kV and MV portal images, can be obtained in either the OBI (On-Board Imager) computer of the treatment machine or the Mosaiq computer next to the treatment machine. It is straightforward to improve our current ADQ programs 1) to check the dynalog file (against the corresponding treatment plan in DICOM format) right after the treatment deliveries, and 2) to include the daily IGRT images, into a single delivery check report, similar to the ViewRay delivery verification report.

# Conclusion

A procedure was developed in this study and implemented in a computer program to automatically verify the ViewRay treatment deliveries and to generate the concise daily treatment reports. The method is useful to facilitate the delivery error detection, and to expedite physician daily IGRT review and physicist weekly chart review.

# Acknowledgement

The project described was partially supported by the AHRQ (Agency for Healthcare Research and Quality) grant number 1 R01 HS022888-01 and its contents are solely the responsibility of the authors and do not necessarily represent the official views of the Agency for Healthcare Research and Quality.

This study is partially supported by a research grant from ViewRay Incorporated.

# References

1. Mutic S, Dempsey JF. The ViewRay System: Magnetic Resonance–Guided and Controlled Radiotherapy. Seminars in Radiation Oncology 2014;24(3):196-9.

2. Bostel T, Nicolay NH, Grossmann JG, et al. MR-guidance--a clinical study to evaluate a shuttle- based MR-linac connection to provide MR-guided radiotherapy. Radiat Oncol 2014;9:12.

3. Goyal S, Kataria T. Image Guidance in Radiation Therapy: Techniques and Applications. Radiology Research and Practice 2014;2014:10.

4. Dempsey JF, Benoit D, Fitzsimmons JR, et al. A Device For Realtime 3D Image-Guided IMRT. International Journal of Radiation Oncology*Biology*Physics 2005;63:S202.

5. Bortfeld T. IMRT: a review and preview. Physics in Medicine and Biology 2006;51(13):R363.

6. Yan D, Vicini F, Wong J, Martinez A. Adaptive radiation therapy. Physics in Medicine and Biology 1997;42(1):123-32.

7. Oh S, Stewart J, Moseley J, et al. Hybrid adaptive radiotherapy with on-line MRI in cervix cancer IMRT. Radiother Oncol 2014 Feb;110(2):323-8.

8. Kutcher GJ, Coia L, Gillin M, et al. Comprehensive QA for radiation oncology: report of AAPM Radiation Therapy Committee Task Group 40. Med Phys 1994 Apr;21(4):581-618.

9. Zhu X, Ge Y, Li T, et al. A planning quality evaluation tool for prostate adaptive IMRT based on machine learning. Med Phys 2011 Feb;38(2):719-26.

10. Childress N, Chen Q, Rong Y. Parallel/Opposed: IMRT QA using treatment log files is superior to conventional measurement-based method. J Appl Clin Med Phys 2015;16(1):5385.

11. Agnew CE, Irvine DM, McGarry CK. Correlation of phantom-based and log file patient-specific QA with complexity scores for VMAT. J Appl Clin Med Phys 2014;15(6).

12. Litzenberg DW, Moran JM, Fraass BA. Verification of dynamic and segmental IMRT delivery by dynamic log file analysis. J Appl Clin Med Phys 2002;3(2):63-72.

13. Dinesh Kumar M, Thirumavalavan N, Venugopal Krishna D, Babaiah M. QA of intensity-modulated beams using dynamic MLC log files. Journal of Medical Physics 2006 Jan-Mar;31(1):36-41.

14. Calvo-Ortega JF, Teke T, Moragues S, Pozo M, Casals-Farran J. A Varian DynaLog file-based procedure for patient dose-volume histogram–based IMRT QA. J Appl Clin Med Phys 2014;15(2):4665.

15. Sun B, Rangaraj D, Palaniswaamy G, et al. Initial experience with TrueBeam trajectory log files for radiation therapy delivery verification. Practical Radiation Oncology 2013.

16. Yang D, Li H, Sun B, Tan J, Mutic S. Direct 3D Fluence Calculation from Machine Beam Parameters for VMAT Delivery Verification. Med Phys 2012;39:3957.

17. Schreibmann E, Dhabaan A, Elder E, Fox T. Patient-specific quality assurance method for VMAT treatment delivery. Med Phys 2009;36(10):4530-5.

18. Rangaraj D, Yang D, Goddu SM, et al. Dynalog Based Quality Assurance for Rapid Arc Therapy. Med Phys 2009;36(6):2575-.

19. Chandraraj V, Stathakis S, Manickam R, et al. Consistency and reproducibility of the VMAT plan delivery using three independent validation methods. J Appl Clin Med Phys 2010;12(1):3373.

20. Wu Y, Mutic S, Rangaraj D, et al. A Software Tool That Automatically, Autonomously, Intelligently and Instantly Verify Patient Radiation Therapy Beam Deliveries. Med Phys 2011;38:3807.

21. Fontenot JD. Feasibility of a remote, automated daily delivery verification of volumetric-modulated arc therapy treatments using a commercial record and verify system. J Appl Clin Med Phys 2012;13(2):3606.

22. Low DA, Harms WB, Mutic S, Purdy JA. A technique for the quantitative evaluation of dose distributions. Medical Physics 1998;25(5):656-61.

23. Li HH, Rodriguez VL, Green OL, et al. Patient-Specific Quality Assurance for the Delivery of 60Co Intensity Modulated Radiation Therapy Subject to a 0.35-T Lateral Magnetic Field. International Journal of Radiation Oncology*Biology*Physics 2015;91(1):65-72.


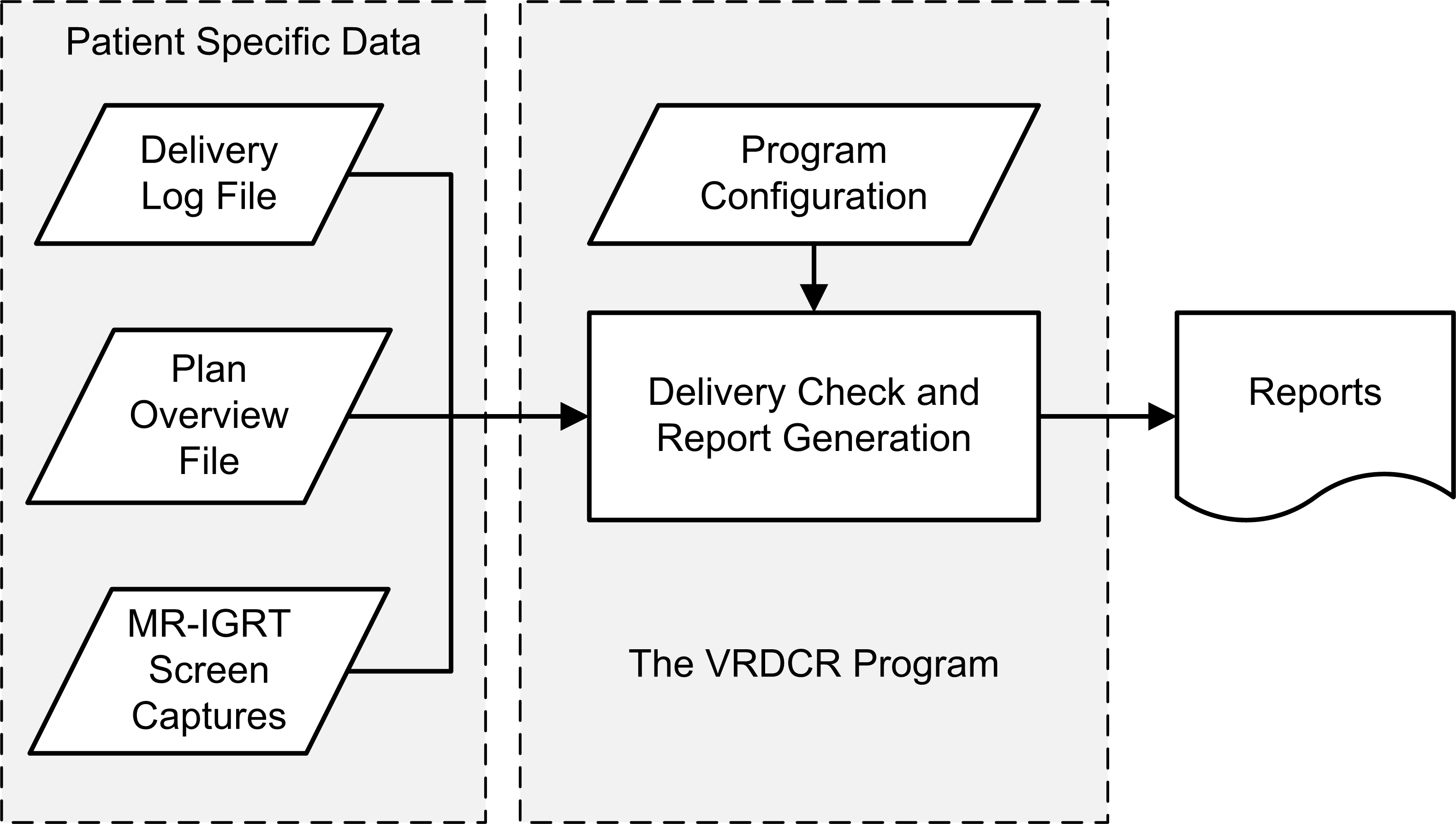


Figure 1: The system workflow


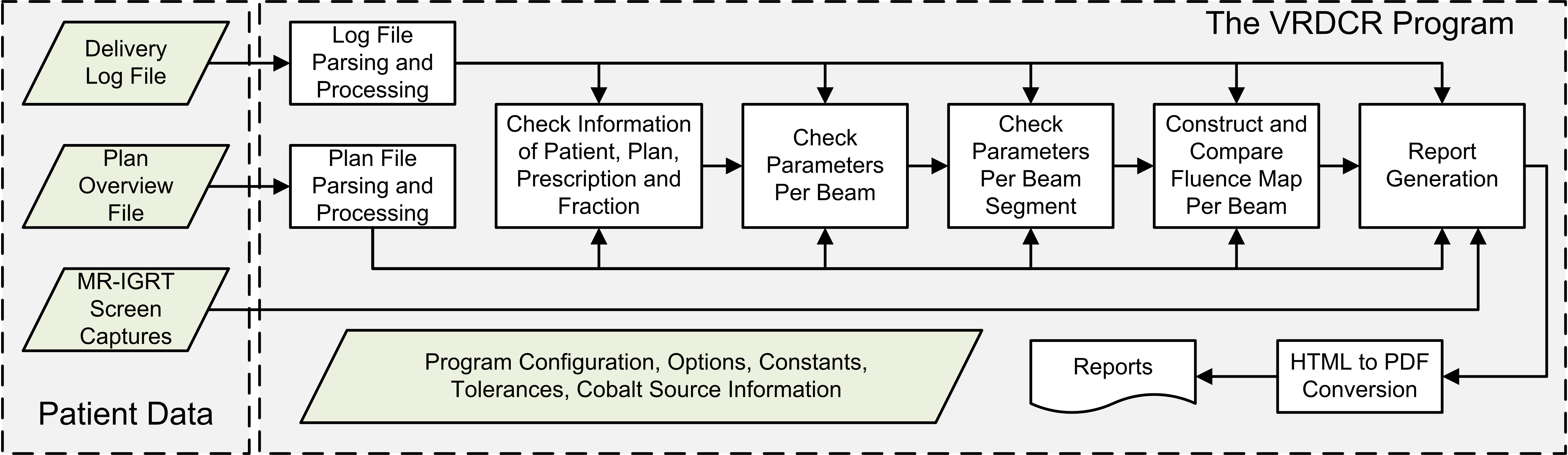


Figure 2: VRDCR program flow chart

Table 1: List of items in categories checked by VRDCR program.

| **Categories** | **Items** |
| --- | --- |
| Patient information | Patient name and ID |
| Prescription information | Prescription dose, PTV target, number of treatment fractions |
| Cobalt-60 source information | Source serial #, calibration date, calibration source strength and dose rate, the decayed source strength and dose rate |
| Treatment plan | Plan name, total number of treatment beams |
| Per beam | Gantry angle, number of segments, total beam-on time, beam fluence intensity map |
| Per segment | MLC leaf positions, beam-on time |

| 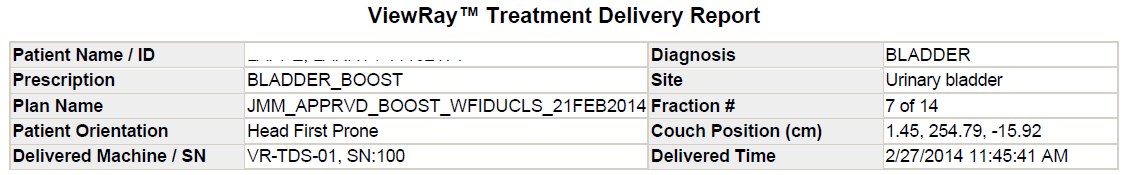  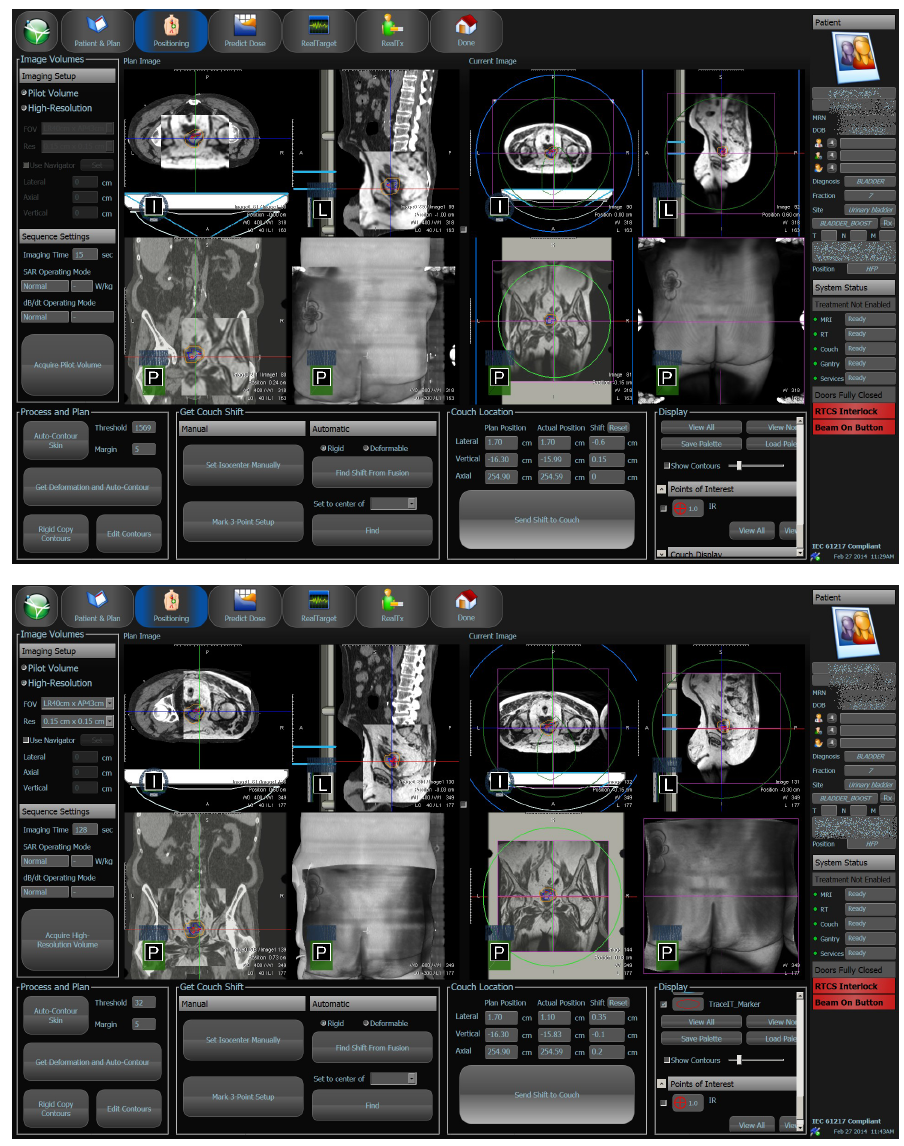 | 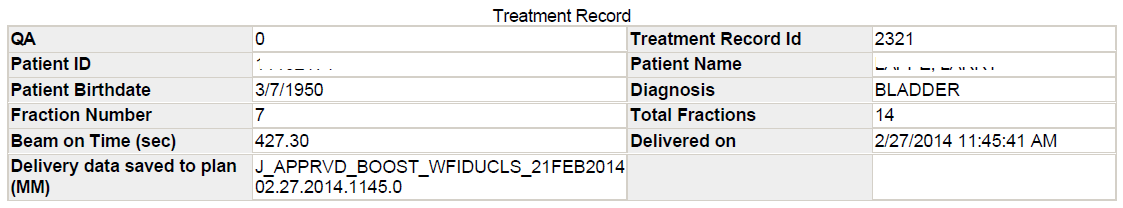  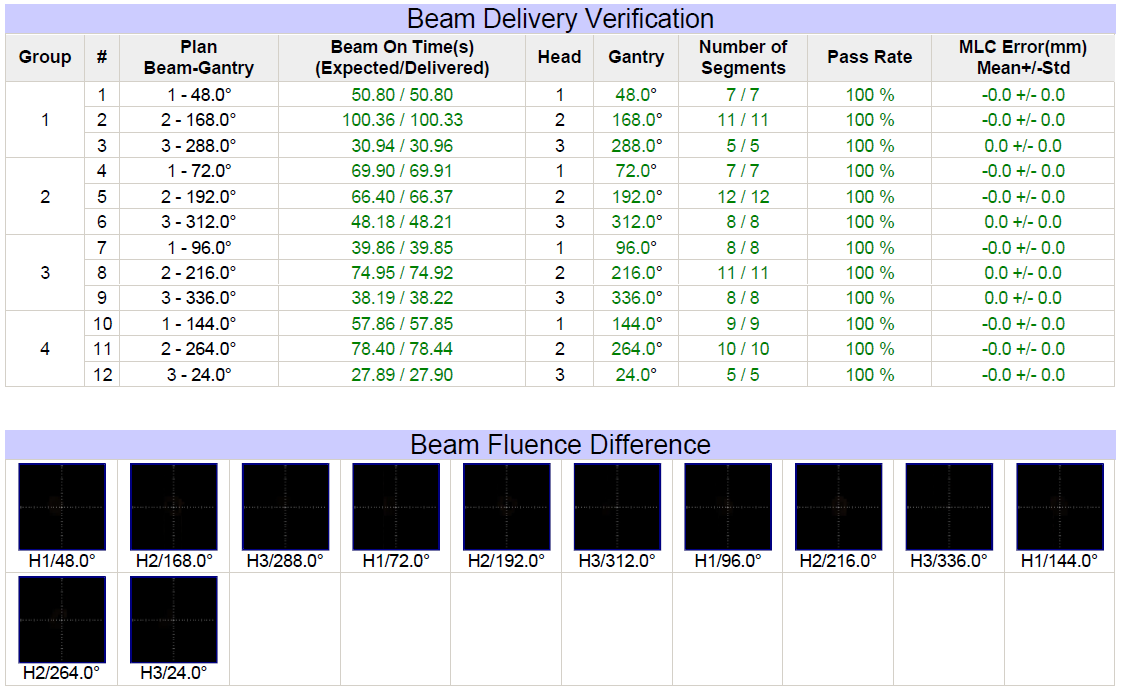  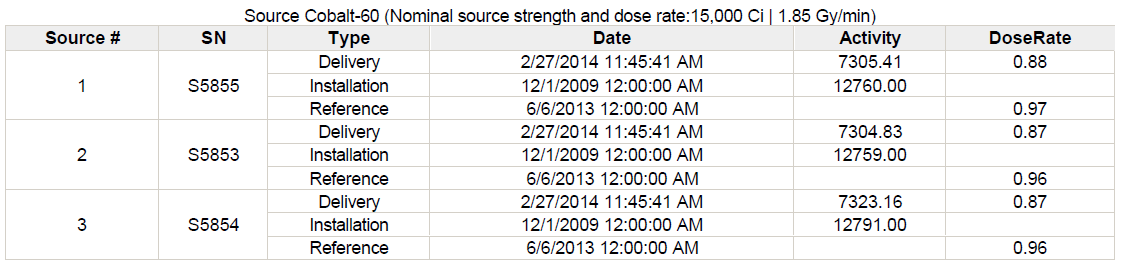 |
| --- | --- |

Figure 3: A patient treatment delivery report. (Left) Plan information and image guidance screen captures. (Right) Beam delivery verification results and the Cobalt source information. The fluence difference maps are all black because there was no significant difference between the beam fluence computed from the planned beam parameters and from the delivered beam parameters.


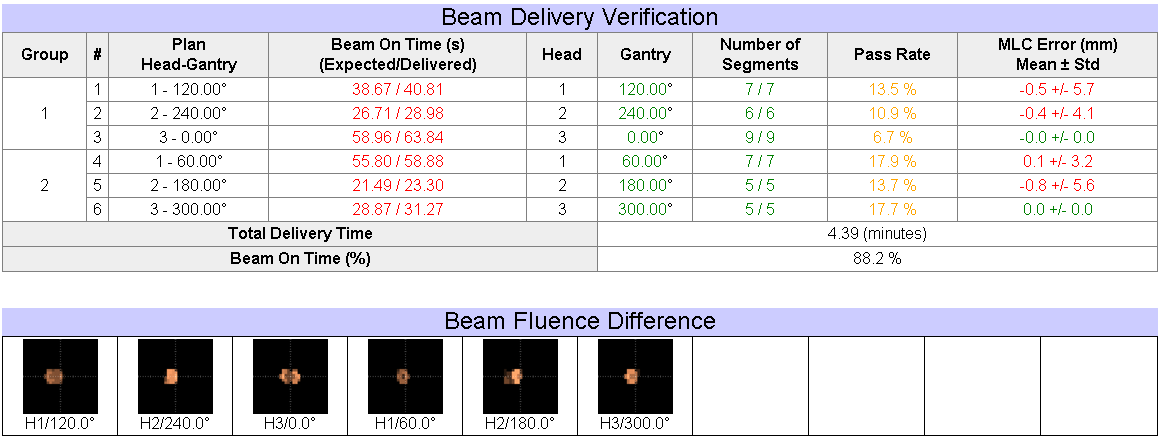


Figure 4: Demonstration of detected treatment delivery errors in an earlier software version test.


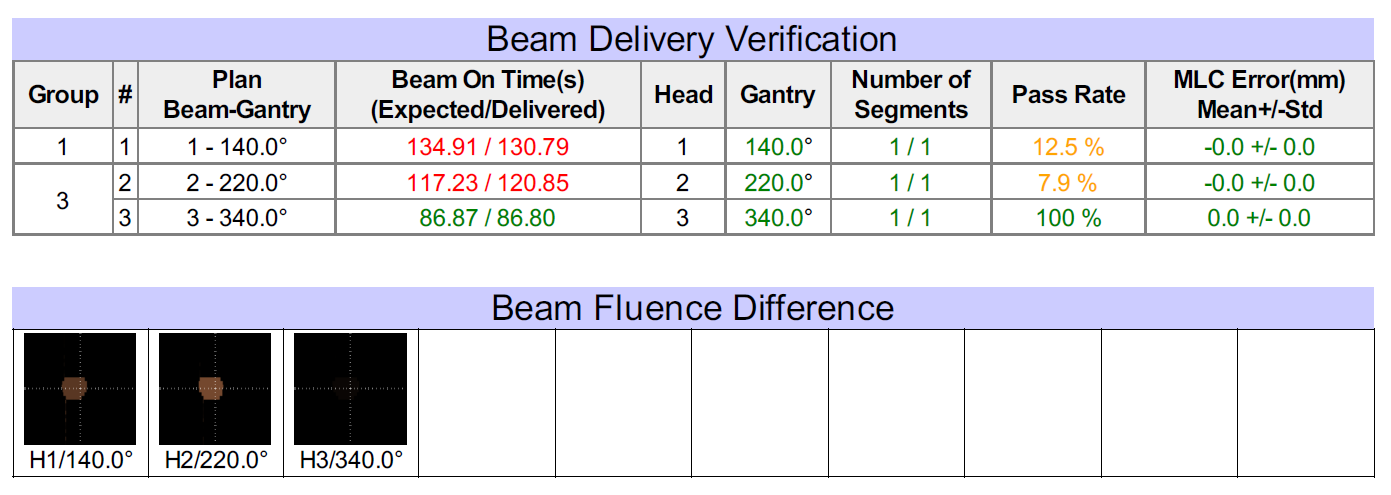


Figure 5: Example of the simulated errors detected by VRDCR in the clinical commissioning tests. In this example, the beam weighting was adjusted by 1% for beams 1 and 2. The errors were reflected as a change in the delivery Beam-On Time which also resulted in a difference in the beam fluence.
